# KYNA Ameliorates Hepatic Ischemia–Reperfusion Injury by Activating the Hippo Signalling Pathway via FTO‐Dependent m6A Demethylation of LATS1

**DOI:** 10.1111/cpr.70048

**Published:** 2025-04-25

**Authors:** Wenjie Zheng, Xiaowen Wang, Haoqi Chen, Kaiming He, Xijing Yan, Yuan Zhang, Yang Yang, Peng Zhang, Wenfeng Zhu, Shuguang Zhu, Hua Li

**Affiliations:** ^1^ Department of Hepatic Surgery, Liver Transplantation The Third Affiliated Hospital of Sun Yat‐Sen University Guangzhou China; ^2^ Guangdong Key Laboratory of Liver Disease Research The Third Affiliated Hospital of Sun Yat‐Sen University Guangzhou China; ^3^ Department of Vascular Surgery Zhejiang Provincial People's Hospital (Affiliated People's Hospital), Hangzhou Medical College Zhejiang Hangzhou China; ^4^ Department of Thyroid and Breast Surgery The Third Affiliated Lingnan Hospital of Sun Yat‐Sen University Guangzhou China; ^5^ Department of Pathology Guangdong Provincial Hospital of Chiese Medicine Guangzhou China; ^6^ Department of Hepatobiliary and Pancreatic Surgery The Second Affiliated Hospital of Guangzhou Medical University Guangzhou China

**Keywords:** FTO, hepatic ischemia–reperfusion injury, Kynurenic acid, liver transplantation, m6A

## Abstract

Hepatic ischemia–reperfusion injury (HIRI) substantially influences the prognosis of liver transplant recipients. Although kynurenic acid (KYNA) has been associated with protective effects against ischemia–reperfusion injury in various organs, the precise mechanisms underlying its protective role in HIRI are not well elucidated. In this study, a 70% mouse HIRI model and an in vitro hypoxia/reoxygenation model were employed to examine the protective effects of KYNA on HIRI. In this study, we illustrate that KYNA influences the methylation status of the Hippo signalling pathway by enhancing the expression of the fat mass and obesity‐associated gene (FTO). Within this pathway, large tumour suppressor kinase 1 (LATS1) is identified as a direct target of FTO. Moreover, the stability of LATS1 mRNA exhibits an inverse correlation with FTO levels and is modulated through its interaction with YTH N6‐Methyladenosine RNA Binding Protein F2 (YTHDF2). The reduction in LATS1 expression facilitated Yes‐associated protein (YAP) nuclear translocation, decreased hepatocyte apoptosis, and mitigated HIRI. Clinically, elevated levels of serum KYNA correlate with a diminished severity of liver injury post‐transplantation. our work revealed that KYNA possesses significant clinical translational potential for the prevention of HIRI, and further exploration of its underlying mechanisms was conducted.

## Graphical abstract

Our study elucidates the cellular and molecular mechanisms underlying KYNA's protective effects against HIRI. It emphasises that KYNA modulates the methylation status of the Hippo signalling pathway through the activation of AHR, thereby mitigating damage by promoting LATS1 degradation and facilitating the nuclear translocation of YAP.

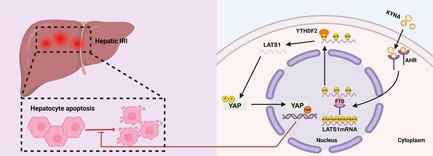

## 1 Introduction

Hepatic ischemia–reperfusion injury (HIRI) predominantly manifests in the context of hemorrhagic shock and post‐operative settings such as liver transplantation and partial liver resection, thereby predisposing patients to early liver dysfunction and graft rejection [1, 2, 3]. HIRI is primarily an aseptic inflammatory response triggered by the re‐establishment of blood flow following ischemic and hypoxic conditions, and is characterised by oxidative stress, apoptosis, and inflammatory dysregulation [4]. Notably, there are currently no clinically approved pharmacological interventions available for the prevention and management of HIRI. Therefore, it is imperative to elucidate the underlying molecular mechanisms of HIRI and to identify novel preventive and therapeutic targets to effectively mitigate its effects.

Kynurenic acid (KYNA), an endogenous metabolite of tryptophan synthesised via the kynurenine pathway, has been documented to participate in anti‐inflammatory and immunomodulatory processes across various organs by activating the G‐protein‐coupled receptor 35 (GPR35) and the aryl hydrocarbon receptor (AHR) [5, 6, 7]. Recent studies have elucidated the involvement of KYNA in the pathophysiology of ischemia–reperfusion injury (IRI) across various organs through multiple mechanistic pathways. Olenchock et al. demonstrated that remote ischemic conditioning augments hepatic KYNA production, thereby conferring cardioprotection against myocardial ischemia/reperfusion (I/R) injury in murine models [8]. Similarly, Nahomi et al. reported that elevated KYNA levels can safeguard retinal ganglion cells from I/R‐induced damage [9]. Furthermore, preclinical evidence indicates that KYNA administration reduces infarct size and enhances behavioural and cognitive outcomes in human stroke patients [10]. Similar to IRI in other solid organs, HIRI involves aseptic inflammation triggered by the restoration of blood flow following ischemia and hypoxia. We hypothesise that KYNA may also exert a protective effect against the HIRI process. However, the role of KYNA in HIRI remains unclear, and elucidating its underlying molecular mechanisms is essential for the development of effective therapeutics to mitigate HIRI.

N6‐methyladenosine (m6A) represents a prevalent and evolutionarily conserved RNA modification that is integral to numerous biological processes and pathologies. This modification is characterised by its dynamic and reversible nature, playing a pivotal role in the post‐transcriptional regulation of gene expression in eukaryotic organisms. It exerts its influence on critical processes such as RNA splicing, translation, and degradation. The functional implications of m6A are mediated through the coordinated actions of m6A‐associated enzymes and proteins, commonly referred to as writers, erasers, and readers [11, 12]. Recent studies have also highlighted the role of m6A modification in regulating IRI in various tissues, including the heart, kidney, and brain [13, 14, 15]. Du et al. were the first to elucidate the significant role of m6A methylation in HIRI [16]. Yu et al. demonstrated that methyltransferase‐like protein 3 (METTL3)‐mediated m6A methyladenosine modification of RNA contributes to the upregulation of phosphoenolpyruvate carboxykinase 1 (PCK1) during HIRI, thereby mitigating hepatocyte apoptosis [17]. However, the potential role of m6A modification in HIRI and its underlying mechanisms remain largely unexplored.

In this study, we explored the critical function of KYNA in HIRI by assessing its impact on methylation modification levels through both in vivo and in vitro experiments. Our findings revealed that KYNA enhances the expression of the demethylase fat mass and obesity‐associated gene (FTO) via AHR, resulting in a decrease in overall methylation levels. Concurrently, methylated RNA immunoprecipitation sequencing (MeRIP‐seq) revealed that elevated FTO expression diminishes the post‐transcriptional m6A modification of large tumour suppressor kinase 1 (LATS1). This reduction in m6A modification compromises the stability and expression of LATS1, thereby facilitating the expression and nuclear translocation of Yes‐associated protein (YAP). Furthermore, in clinical specimens, we identified a negative correlation between postoperative KYNA levels and the extent of liver function impairment following transplantation. Additionally, a significant association was observed between the KYNA‐activated Hippo signalling pathway and the degree of liver function deterioration post‐transplantation. These findings suggest that KYNA may have potential therapeutic applications for enhancing liver function in patients after liver transplantation.

## 2 Materials and Methods

### 2.1 Clinical Liver Graft Sample

From January 2021 to December 2023, we collected liver samples from 30 donors during orthotopic liver transplantation. Inclusion criteria: (a) Patients aged between 18 and 75 years (Irrespective of gender); (b) Patients undergoing liver transplant surgery due to liver failure; (c) Patients with no prior history of tumour treatment. Exclusion criteria: (a) Patients requiring liver transplant surgery due to malignant tumours; (b) Donor livers exhibiting significant fatty degeneration. Liver samples were partially embedded in paraffin for immunohistochemical analysis, while the remaining portions were preserved in liquid nitrogen for subsequent western blot (WB) analysis. Reperfusion specimens were obtained within 2 h following hepatic artery anastomosis. Additionally, serum ALT and AST levels were monitored from POD 1 to POD 5, and serum KYNA concentrations were measured on the first postoperative day. Organ donation was conducted voluntarily, with informed consent obtained from all patients or their relatives.

### 2.2 Animals

The experimental subjects comprised mice with a C57BL/6 genetic background, procured from the Model Animal Research Center of Nanjing University (Nanjing, China). Liver‐specific YAP knockdown (*YAP‐LKD*) mice were generated by crossing albumin‐Cre (Alb‐Cre) and YAP^flox/flox^ mice at the Model Animal Research Center of Nanjing University (Nanjing, China). All animals were maintained in a specific pathogen‐free (SPF) environment with a controlled temperature of 25°C, humidity levels ranging from 40% to 70%, a 12‐h light–dark cycle, and ad libitum access to food and water. All animal experiments were conducted in strict compliance with the ARRIVE (Animal Research: Reporting of In Vivo Experiments) guidelines to ensure transparency, reproducibility, and ethical integrity. The study design, animal allocation, sample size determination, and statistical methods were established following ARRIVE 2.0 recommendations. Kynurenic acid was administered intraperitoneally at a dosage of 20 mg/kg for a duration of 14 days. At the end of the study, mice were euthanized using sodium pentobarbital (100 mg/kg, intraperitoneal injection), ensuring a humane and ethical endpoint. Death was confirmed by the cessation of heartbeat and respiratory movement before tissue collection.

### 2.3 Cell Culture

The cell hypoxia‐reoxygenation (H/R) model involved incubating THLE‐2 cells and primary mouse hepatocytes (PMHs) within a humidified sealed chamber under hypoxic conditions (5% CO_2_, 1% O_2_, and 94% N_2_) for a duration of 6 h, utilising a glucose‐free and serum‐free Dulbecco's Modified Eagle Medium (DMEM). Following this, the cells underwent reoxygenation for 8 h at room temperature in a normoxic environment (95% O_2_ and 5% CO_2_) with a complete medium. To modulate the expression levels of AHR, FTO, YTHDF2, and LATS1, small interfering RNA (siRNA) and plasmids sourced from GeneCopoeia (Rockville, MD, USA) were utilised. These genetic materials were transfected using Lipofectamine 3000 (Invitrogen, Carlsbad, CA, USA) according to the manufacturer's instructions. For pharmacological intervention, THLE‐2 cells and PMHs were treated with KYNA (10 μM, Selleck Chemicals, Houston, TX, USA) for 30 min prior to H/R.

### 2.4 Total RNA Extraction, mRNA Stability Assay, and RT‐qPCR Analysis

Total RNA was extracted from frozen samples and cultured cells using TRIzol reagent (Invitrogen, 15596018) according to the manufacturer's protocol. For mRNA stability assays, cells were cultured in 6‐well plates and treated with actinomycin D (MCE, HY‐17559) at a final concentration of 5 μg/mL. Cells were harvested at 0, 1, 2, 3, 4, and 5 h post‐incubation. Total RNA was extracted and subjected to reverse transcription quantitative polymerase chain reaction (RT‐qPCR) to assess the relative expression levels of LATS1 mRNA, normalised to the 0‐h time point. The mRNA expression of target genes was normalised to β‐actin and calculated using the 2^−ΔΔCt^ method. The primer sequences used are listed in Table S1.

### 2.5 RNA Pulldown Assays

RNA pulldown assays were conducted in accordance with the Pierce Magnetic RNA‐Protein Pulldown Kit protocol. RNA was prepared via in vitro transcription and labelled with biotin. Poly(A)25 RNA served as a negative control. Following the extraction of total protein from each experimental group, RNA pulldown assays were executed according to the standardised protocol, and protein expression levels were verified through WB analysis.

### 2.6 Statistical Analysis

Statistical analyses and data visualisation were conducted utilising GraphPad Prism 8 software (GraphPad Software, San Diego, CA). Qualitative data were derived from a minimum of three independent replicates. Quantitative data are presented as the mean ± standard deviation. For single comparisons, an unpaired two‐tailed *t*‐test was employed. A one‐way analysis of variance (ANOVA) was applied to assess statistical significance between groups, followed by Dunnett's post hoc test for multiple comparisons against the control group. Linear regression analysis was utilised to examine relationships, with a *p* value < 0.05 considered indicative of statistical significance.

## 3 Results

### 3.1 KYNA Mitigates HIRI in Mice

**FIGURE 1:**
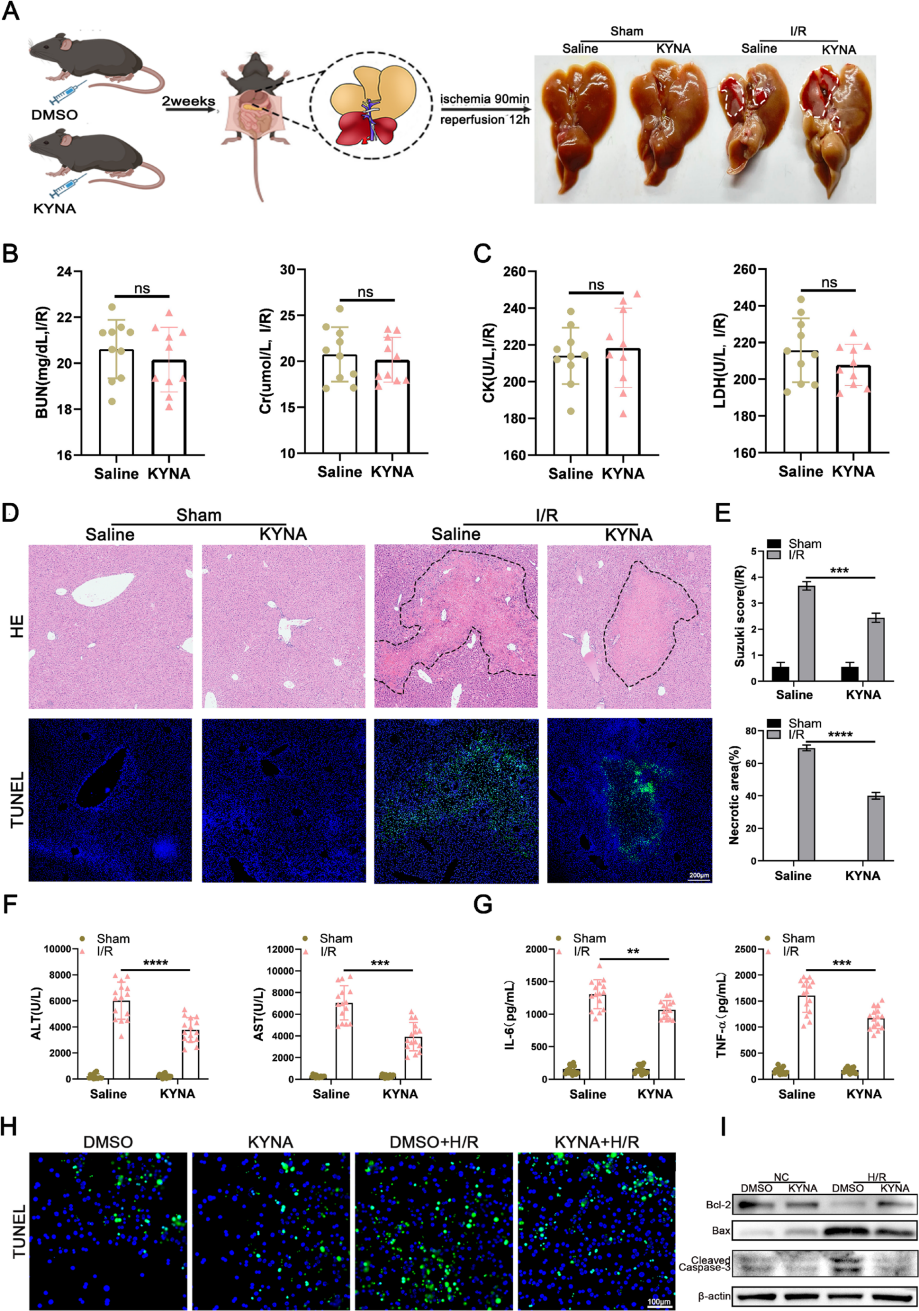
KYNA mitigates HIRI in mice. (A) Schematic diagram for animal model protocols. The area circled by white dashed line represent injured area. (B) Serum levels of BUN and Cr were determined (*n* = 10). (C) Serum levels of CK and LDH were determined (*n* = 10). (D) Liver samples were collected from mice that received intraperitoneal injections of saline and KYNA in either the sham group or the I/R group. HE staining and fluorescent TUNEL staining was analysed microscopically (*n* = 15). The area circled by black dashed line represent injured area, Scale bar = 200 μm. (E) Suzuki score and necrosis area were examined. (F) Serum levels of ALT and AST were determined (*n* = 15). (G) Serum levels of IL‐6 and TNF‐α were determined (*n* = 15). (H) The representative TUNEL staining was microscopically analysed in primary mouse hepatocytes isolated from mice with different treatments, Scale bar = 100 μm. (I) The expression of BCL2, cleaved caspase‐3 and BAX in primary mouse hepatocytes isolated from mice in different groups were determined by WB analysis. Each experiment was independently performed more than twice. All results are presented as mean ± SD and statistical significance was assessed using a 2‐tailed Student *t* test. *p* < 0.05 was considered statistically significant, ns no significance.

To explore the involvement of KYNA in HIRI, we initially pretreated mice with intraperitoneal injections of KYNA at varying concentrations, followed by the induction of a 70% hepatic I/R model (Figure 1A). The results indicated that KYNA at a concentration of 20 mg/kg exhibited the most pronounced protective effect against HIRI in mice. At this concentration, KYNA did not significantly impact body weight, respiration, perioperative mortality, or anaesthesia recovery time in the mice (Figure S1A,B and Table S2). The ELISA revealed no significant impact of KYNA on cardiac and renal function in mice (Figure 1B,C). In contrast, KYNA significantly ameliorated HIRI and reduced hepatocyte apoptosis and liver injury in mice compared to the control group (Figure 1D–G). Furthermore, experiments on primary mouse hepatocytes corroborated these findings, showing a significant decrease in the number of apoptotic cells subsequent to the application of KYNA (Figure 1H). WB analysis of primary mouse hepatocytes demonstrated that the administration of KYNA significantly decreased the levels of pro‐apoptotic proteins BAX and cleaved caspase‐3, and notably elevated the expression of anti‐apoptotic protein BCL2 (Figure 1I).

### 3.2 KYNA Alleviates HIRI by Activating AHR

**FIGURE 2:**
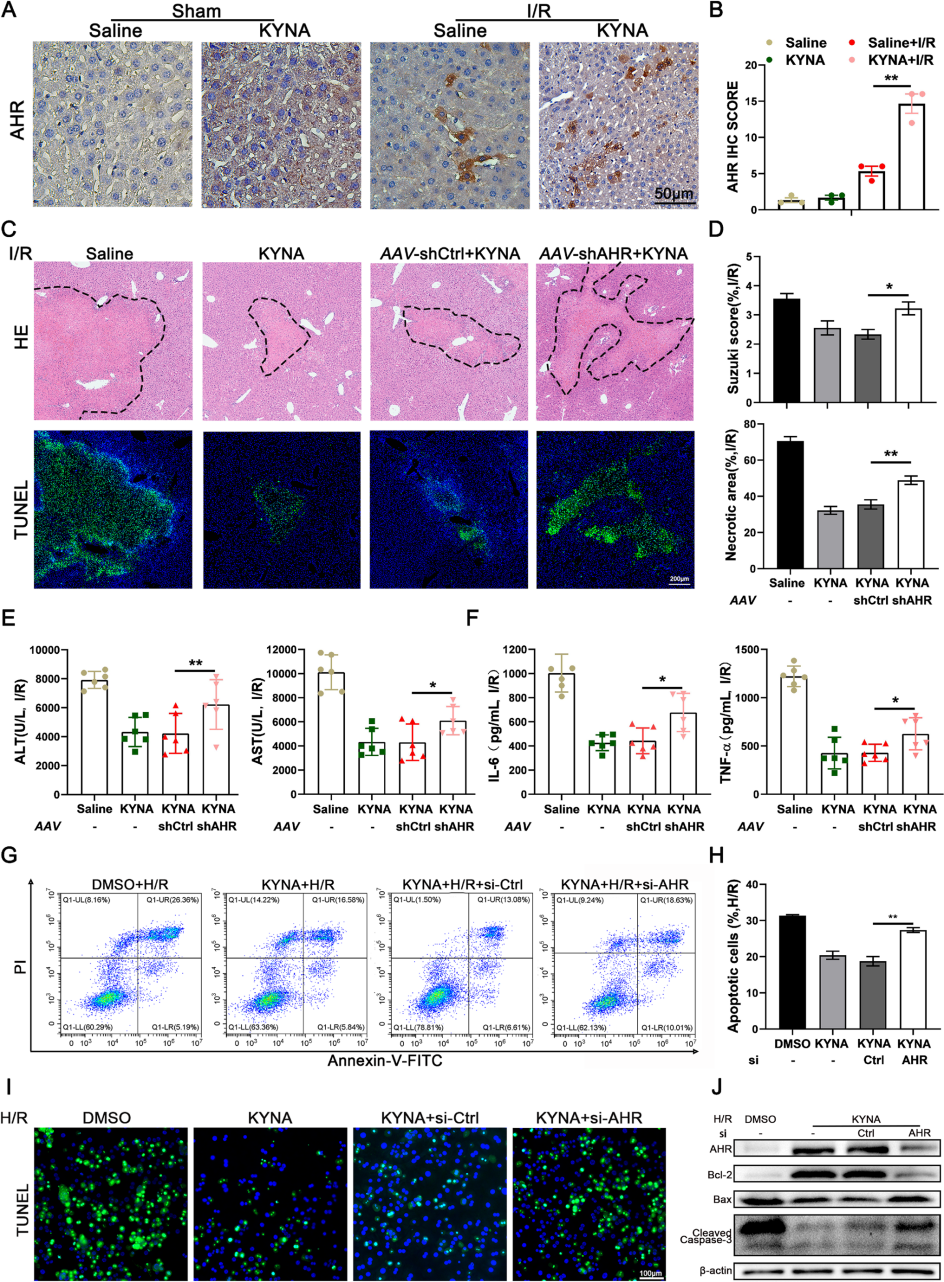
KYNA alleviates HIRI by activating AHR. (A) Liver samples were collected from different treated mice. The expression of AHR was detected by immunohistochemical staining, Scale bar = 50 μm. (B) Relative quantification of IHC staining of AHR in liver tissues. (C) Liver samples were collected from different treated mice. HE staining and fluorescent TUNEL staining was analysed microscopically (*n* = 6). The area circled by black dashed line represent injured area, Scale bar = 200 μm. (D) Suzuki score and necrosis area were examined. (E) Serum levels of ALT and AST were determined (*n* = 6). (F) Serum levels of IL‐6 and TNF‐α were determined (*n* = 6). (G) Representative flow cytometry map of the proportion of early and late apoptotic cells in THLE‐2 cells after different treatments (*n* = 3). (H) The apoptosis rate of THLE‐2 cells after different treatments were examined. (I) The representative TUNEL staining was microscopically analysed in primary mouse hepatocytes isolated from mice with different treatments, Scale bar = 100 μm. (J) The expression of BCL2, cleaved caspase‐3 and BAX in primary mouse hepatocytes isolated from mice in different groups were determined by WB analysis. Each experiment was independently performed more than twice. All results are presented as mean ± SD and statistical significance was assessed using a 2‐tailed Student *t* test. *p* < 0.05 was considered statistically significant.

To further explore the mechanism by which KYNA alleviates HIRI, we examined several important molecular targets at which it functions. WB and RT‐qPCR analyses revealed no significant differences in the expression levels of GPR35 protein and mRNA following KYNA pretreatment compared to the control group. However, a significant upregulation of AHR was observed (Figure S1C,D), a finding corroborated by immunohistochemical staining (Figure 2A,B). To further investigate the role of KYNA‐activated AHR in HIRI, we employed adeno‐associated virus to inhibit AHR expression in mouse liver tissue. The results indicated that the reduction of hepatic AHR levels significantly diminished the protective effect of KYNA on HIRI in mice (Figure 2C–F). Consistent findings were observed in cell‐based experiments, where decreased AHR expression significantly reduced KYNA's protective effect on hepatocytes. Flow cytometry analysis of THLE‐2 cells demonstrated that reduced AHR expression markedly increased the apoptosis rate (Figure 2G,H). Furthermore, experiments on primary mouse hepatocytes corroborated these findings, showing a significant increase in the number of apoptotic cells following AHR downregulation (Figure 2I). WB analysis of primary mouse hepatocytes revealed that the reduced expression of AHR was associated with a significant increase in the levels of the pro‐apoptotic proteins BAX and cleaved caspase‐3, and a significant decrease in the expression of the anti‐apoptotic protein BCL2 (Figure 2J). These findings suggest that KYNA confers protection against HIRI through the activation of AHR.

### 3.3 m6A Modification and FTO Are Involved in KYNA‐Mediated HIRI Protection

**FIGURE 3:**
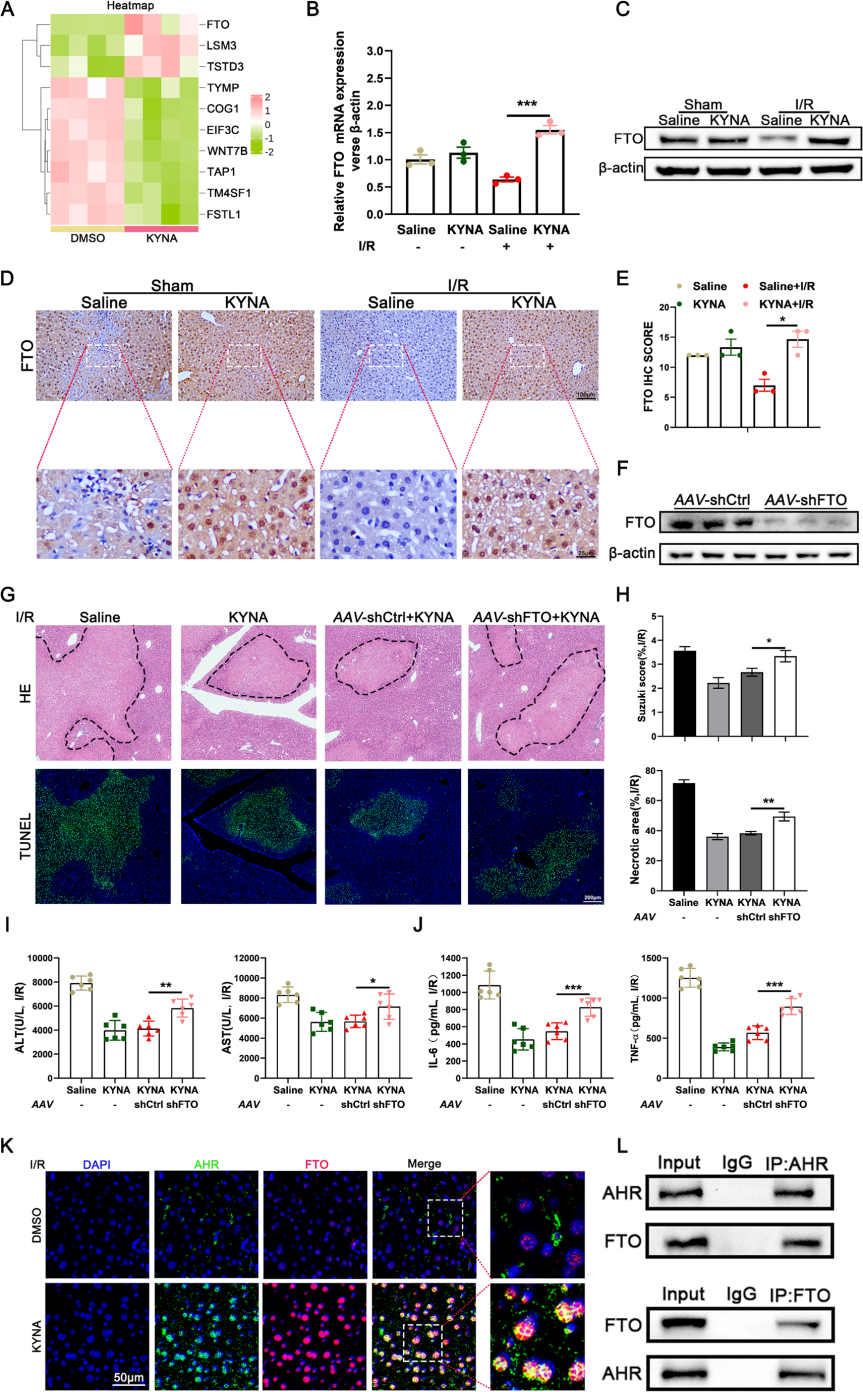
m6A modification and FTO are involved in KYNA‐mediated HIRI protection. (A)Transcriptome sequencing of THLE‐2 cells (*n* = 4). (B) RT‐qPCR analysis of FTO in mouse liver tissues (*n* = 3). (C) The expression of FTO in mouse liver tissues with different treatment was determined by WB analysis. (D) Liver samples were collected from different treated mice. The expression of FTO was detected by immunohistochemical staining, Scale bar = 100 μm. (E) Relative quantification of IHC staining of FTO in liver tissues. (F) FTO protein levels detected by WB in the livers from AAV‐shCtrl and AAV‐shFTO mice (*n* = 6). (G) Liver samples were collected from different treated mice. HE staining and fluorescent TUNEL staining was analysed microscopically (*n* = 6). The area circled by black dashed line represent injured area, Scale bar = 200 μm. (H) Suzuki score and necrosis area were examined. (I) Serum levels of ALT and AST were determined (*n* = 6). (J) Serum levels of IL‐6 and TNF‐α were determined (*n* = 6). (K) Immunofluorescence staining of AHR (green fluorescence) and FTO (red fluorescence). Nuclei were counterstained with DAPI (blue fluorescence), Scale bar = 50 μm. (L) THLE‐2 cells were subjected to immunoprecipitation using an anti‐AHR antibody and anti‐FTO antibody. Each experiment was independently performed more than twice. All results are presented as mean ± SD and statistical significance was assessed using a 2‐tailed Student *t* test. *p* < 0.05 was considered statistically significant, ns no significance.

To elucidate the downstream regulators of KYNA that confer protection in HIRI through their activation, we conducted RNA‐sequencing analysis on THLE‐2 cells. The analysis revealed a significant upregulation of demethylase FTO expression following KYNA pretreatment (Figure 3A). Subsequently, we quantified the expression levels of FTO protein and RNA in murine liver tissue. During IRI, the expression of FTO was markedly elevated following KYNA treatment (Figure 3B,C). Immunohistochemical analysis corroborated these findings (Figure 3D,E). To investigate the impact of KYNA on m6A modification during HIRI, we quantified the m6A modification levels in RNA extracted from mouse liver tissue and THLE‐2 cells post‐KYNA pretreatment. The m6A modification levels were significantly increased following I/R and H/R, but were notably reduced after KYNA treatment (Figure S1E). To elucidate the role of FTO in KYNA‐mediated HIRI protection, adeno‐associated virus was employed to inhibit FTO expression in murine liver tissue. The efficient knockdown of FTO in livers was confirmed by WB analysis of the liver tissue (Figure 3F). The findings indicated that the reduction in hepatic FTO expression markedly diminished the protective effect of KYNA on HIRI in mice (Figure 3G–J). Additionally, experiments conducted on primary mouse hepatocytes demonstrated that decreased FTO expression significantly elevated the number of apoptotic hepatocytes (Figure S1F). WB analysis of primary mouse hepatocytes corroborated these findings, revealing a significant upregulation in the expression of pro‐apoptotic proteins BAX and cleaved caspase‐3, alongside a marked downregulation of the anti‐apoptotic protein BCL2 following the suppression of FTO expression (Figure S1G). In liver tissues and cells subjected to FTO knockdown, the reduction in m6A modification levels induced by KYNA was reversed, indicating that KYNA modulates m6A methylation levels by affecting FTO expression. This suggests that FTO may serve as a critical target for KYNA in the regulation of HIRI (Figure S1H). Colocalization assays demonstrated that KYNA treatment elevated the expression levels of AHR and FTO and facilitated the translocation of AHR from the cytoplasm to the nucleus, culminating in intranuclear co‐expression of AHR and FTO (Figure 3K). Co‐immunoprecipitation analysis suggested a potential protein–protein interaction between AHR and FTO following KYNA treatment (Figure 3L). Consequently, we propose that the hepatoprotective effect of KYNA may be mediated via the AHR‐FTO signalling axis during HIRI.

### 3.4 KYNA Mitigates HIRI by Modulating the m6A Modification Within the Hippo Signalling Pathway

**FIGURE 4:**
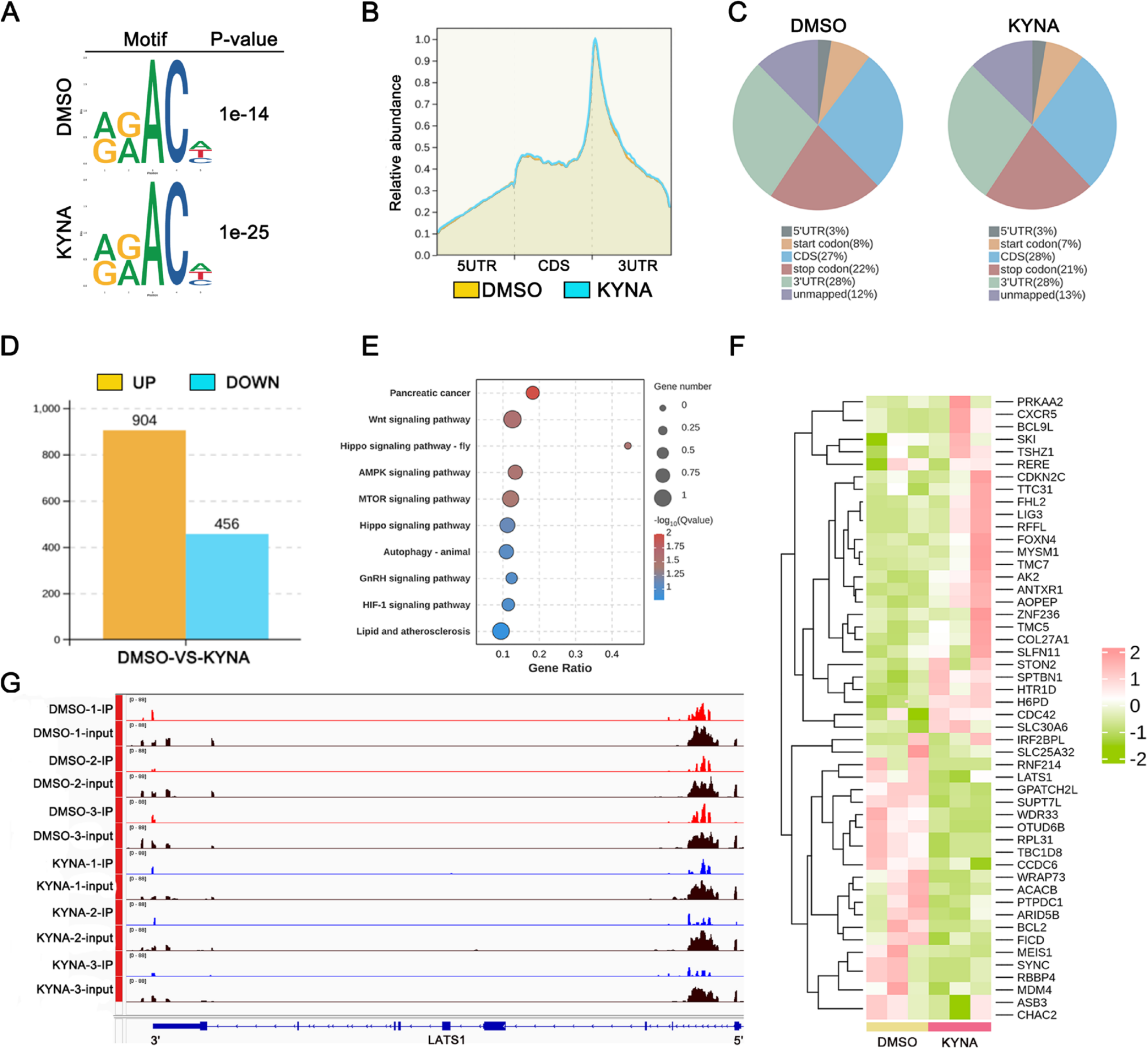
KYNA mitigates HIRI by modulating the m6A modification within the Hippo signalling pathway. (A) Predominant motif identified with m6Aseq peaks in H/R‐DMSO group and H/R‐KYNA group. Motif enrichment *p*‐value was calculated based on Fisher's Exact Test. (B) Distribution of m6A peaks across mRNA in H/R‐DMSO group or H/R‐KYNA group according to m6A RNA sequencing. (C) Pie chart showing the distribution and relative proportion of differential peaks between the H/R‐DMSO group and H/R‐KYNA group according to m6A RNA sequencing. (D) Bar chart show 904 upregulated expression genes and 456 downregulated expression genes with m6A peak change in H/R‐KYNA group compared with H/R‐DMSO group. (E) Kyoto Encyclopedia of Genes and Genomes (KEGG) enrichment analysis showing the enriched signalling pathways. (F) Heatmap of MeRIP‐seq analysis showing differentially expressed genes. (G) KYNA decreased m6A modification of LATS1 mRNA in H/R‐KYNA group.

To identify the direct m6A‐modified targets of KYNA mediated by FTO, we conducted MeRIP‐seq on KYNA‐treated THLE‐2 cells, with dimethyl sulfoxide (DMSO)‐treated cells serving as the control group. MeRIP‐seq analysis revealed that m6A modifications predominantly occur within the consensus sequences 5′‐DRACH‐3′ and 5′‐RRACH‐3′ (where D = G/A/U, *R* = G/A, and H = A/U/C) (Figure 4A, Figure S1I). Notably, m6A peaks are particularly enriched around the 3' untranslated region (3′UTR) and coding sequence (CDS) regions (Figure 4B,C). MeRIP‐seq analysis identified 904 genes exhibiting up‐regulation and 456 genes exhibiting down‐regulation in the KYNA group relative to the DMSO group (Figure 4D). Gene Ontology (GO) enrichment analysis of the MeRIP‐seq data indicated that genes with modified methylation patterns were predominantly involved in metabolism, cell cycle regulation, and immune responses (Figure S1J). Furthermore, pathway mapping of the same dataset using the Kyoto Encyclopedia of Genes and Genomes (KEGG) revealed a significant enrichment in the Hippo signalling pathway (Figure 4E). To further evaluate the extent of m6A methylation modification in the corresponding gene, we selected the differentially expressed gene and observed a significant alteration in the methylation modification of LATS1 within the Hippo signalling pathway (Figure 4F). Analysis of the MeRIP‐seq data revealed the presence of an m6A peak near the CDS region of LATS1 mRNA in the DMSO group, which was diminished in the KYNA group (Figure 4G). Consequently, we hypothesise that LATS1 is a potential target of KYNA, mediated through the modulation of FTO expression.

### 3.5 LATS1 is a Potential Target of KYNA to Regulate Methylation Modifications

**FIGURE 5:**
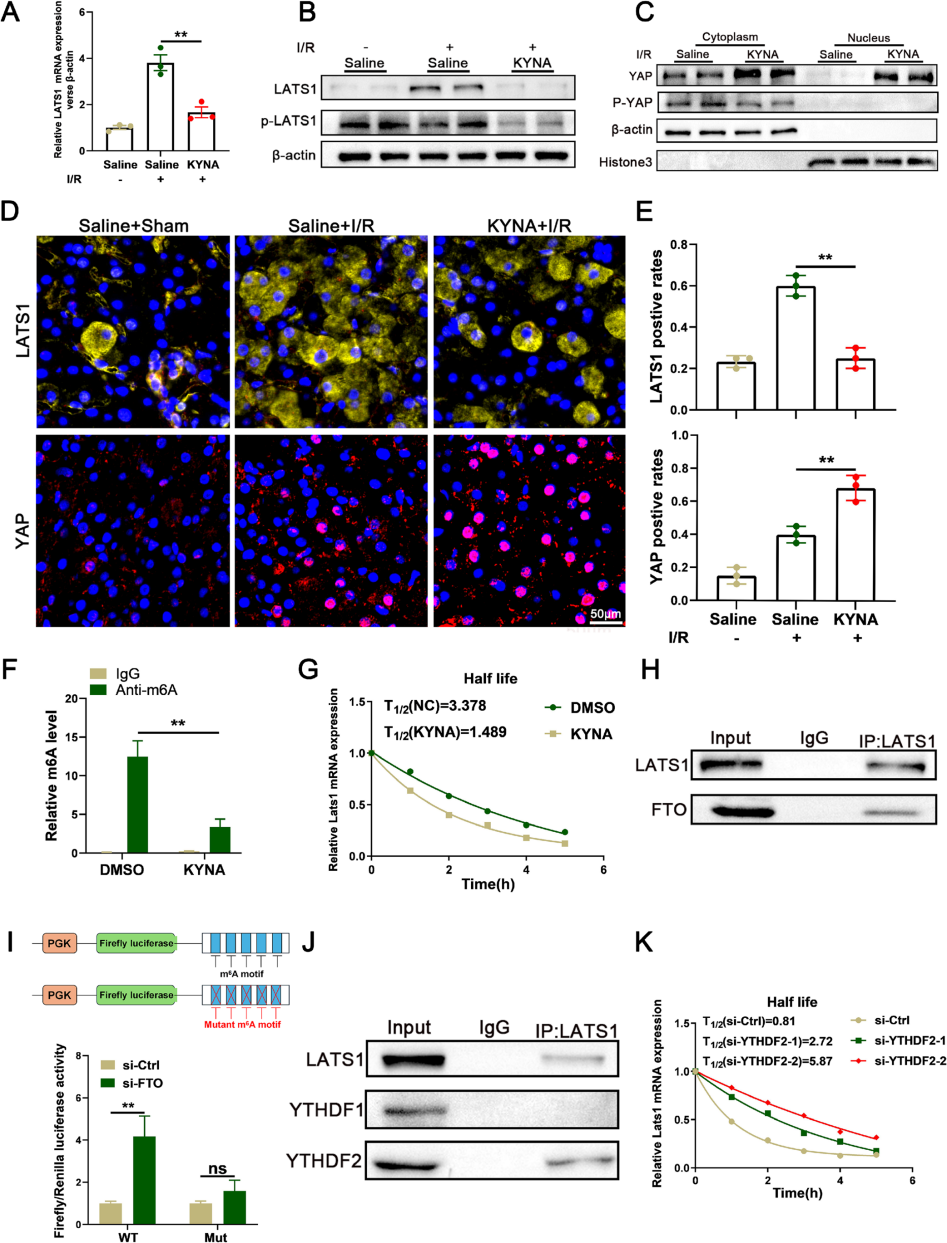
LATS1 is a potential target of KYNA to regulate methylation modifications. (A) RNA level of LATS1 in IRI‐Saline group and IRI‐KYNA group was examined by RT‐qPCR (*n* = 3). (B) Protein level of LATS1, p‐LATS1 in IRI‐Saline group and IRI‐KYNA group was examined by WB. (C) The expression YAP and p‐YAP of cytosolic and nuclear‐enriched fractions in mouse in IRI‐Saline group and IRI‐KYNA group was examined by WB analysis. β‐actin and Histone 3 were used as loading controls of cytosolic and nuclear fractions respectively. (D) Immunofluorescence staining of LATS1 and YAP in liver tissues. Nuclei were counterstained with DAPI, Scale bar = 50 μm. (E) Relative quantification of IF staining of LATS1 and YAP in liver tissues. (F) m6A modification of LATS1 mRNA was detected by MeRIP‐qPCR analysis using anti‐IgG and anti‐m6A antibodies in THLE‐2 cells (*n* = 3). (G) H/R‐KYNA group and H/R‐DMSO group THLE‐2 cells were treated with actinomycin D and harvested at 0, 1, 2, 3, 4, and 5 h. RNA decay rate was determined to estimate the stability of LATS1 mRNA (normalised to the expression at 0 h) (*n* = 3). (H) RNA‐pull down assay showing the interaction between FTO and LATS1 mRNA. (I) Relative luciferase activity of LATS1 mRNA coding sequences with either wild‐type or mutant m6A sites in THLE‐2 cells co‐transfected with si‐FTO or si‐Ctrl (*n* = 3). (J) RNA‐pull down assay showing the interaction between YTHDF2 and LATS1 mRNA. (K) si‐Ctrl group and si‐YTHDF2 group THLE‐2 cells were treated with actinomycin D and harvested at 0, 1, 2, 3, 4, and 5 h. RNA decay rate was determined to estimate the stability of LATS1 mRNA (normalised to the expression at 0 h) (*n* = 3). Each experiment was independently performed more than twice. All results are presented as mean ± SD and statistical significance was assessed using a 2‐tailed Student *t* test. *p* < 0.05 was considered statistically significant, ns no significance.

WB, RT‐qPCR, and immunofluorescence assays were employed to assess the expression levels of LATS1 in mouse liver tissues and THLE‐2 cells. The findings indicated a significant reduction in LATS1 expression following pretreatment with KYNA, relative to the control group. This reduction was concomitant with an increase in YAP expression and a notable nuclear translocation (Figures 5A–E and S2A–E). Subsequently, methylated RNA immunoprecipitation (MeRIP) was conducted using an anti‐m6A antibody and an IgG antibody as a control. The co‐immunoprecipitation with the anti‐m6A antibody resulted in a substantial enrichment of LATS1 mRNA levels compared to the IgG antibody (Figure 5F). These findings indicate that m6A modification plays a role in the regulation of LATS1 mRNA by KYNA. Previous research has demonstrated that the demethylase FTO can destabilise mRNA by reducing m6A methylation modifications [18], Therefore, we propose that KYNA diminishes the stability of LATS1 mRNA by upregulating FTO expression. To test this hypothesis, the mRNA levels of LATS1 were measured at 0, 1, 2, 3, 4, and 5 h following RNA polymerase inhibition via actinomycin D treatment. RT‐qPCR analysis revealed a reduction in the half‐life of LATS1 mRNA post‐KYNA treatment, suggesting that KYNA decreases the stability of LATS1 mRNA (Figure 5G). Using an RNA pull‐down assay and WB, we validated the interaction between LATS1 mRNA and FTO (Figure 5H).

Subsequently, we integrated data from MeRIP‐seq and the RMBase database to identify potential m6A modification sites within LATS1 mRNA. Our analysis revealed that these potential m6A modification sites in the CDS were proximal to the 5′ untranslated region (UTR) (Figure S2F). To verify the accuracy of these predicted modification sites, we constructed luciferase reporter vectors containing both the wild‐type (WT) and mutant LATS1 mRNA coding sequences. We observed that FTO knockdown led to an increase in luciferase activity in the WT group, while no significant effect was noted in the mutant group (Figure 5I). This finding implies that the potential m6A site on LATS1 mRNA is subject to FTO‐mediated demethylation.

To identify which m6A readers directly recognise m6A modification sites and regulate LATS1 expression, we conducted RNA pull‐down assays and WB analyses. Our results indicated that YTHDF2 serves as the m6A reader for LATS1 mRNA (Figure 5J). Previous studies have demonstrated that YTHDF2 facilitates the degradation of its target mRNAs by recognising m6A modifications [19]. Treatment with actinomycin D inhibited mRNA synthesis and revealed that the knockdown of YTHDF2 markedly enhanced the stability of LATS1 mRNA (Figure 5K). WB analyses indicated an inverse correlation between the protein expression levels of LATS1 and YTHDF2 (Figure S2G,H).

### 3.6 KYNA Ameliorates HIRI Through the Activation of the Hippo Signalling Pathway

**FIGURE 6:**
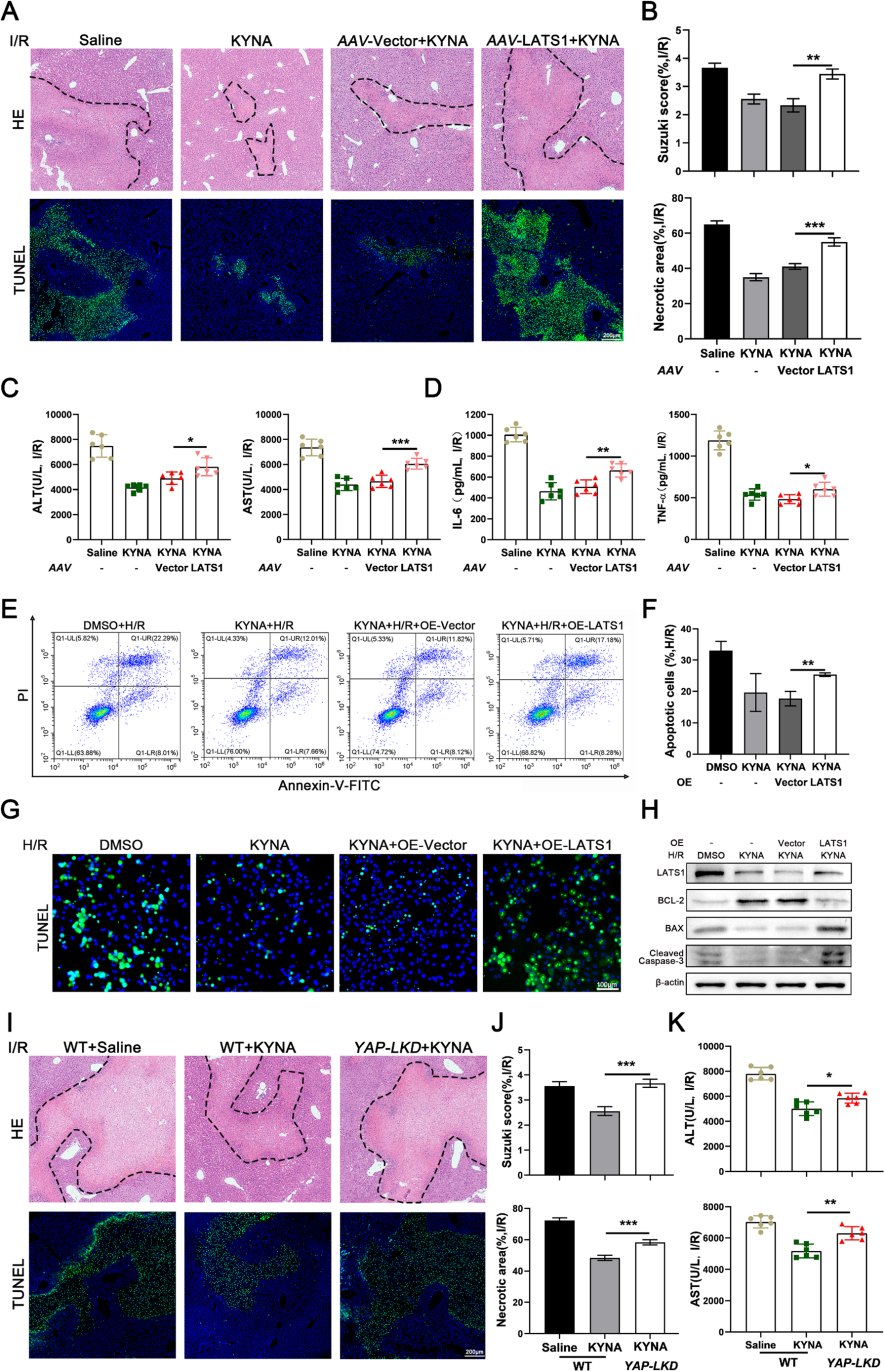
KYNA ameliorates HIRI through the activation of the Hippo signalling pathway. (A) Liver samples were collected from different treated mice. HE staining and fluorescent TUNEL staining was analysed microscopically (*n* = 6). The area circled by black dashed line represent injured area, Scale bar = 200 μm. (B) Suzuki score and necrosis area were examined. (C) Serum levels of ALT and AST were determined (*n* = 6). (D) Serum levels of IL‐6 and TNF‐α were determined (*n* = 6). (E) Representative flow cytometry map of the proportion of early and late apoptotic cells in THLE‐2 cells after different treatments (*n* = 3). (F) The apoptosis rate of THLE‐2 cells after different treatments were examined. (G) The representative TUNEL staining was microscopically analysed in primary mouse hepatocytes isolated from mice with different treatments, Scale bar = 100 μm. (H) The expression of BCL2, cleaved caspase‐3 and BAX in primary mouse hepatocytes isolated from mice in different groups were determined by WB analysis. (I) Liver samples from WT mice and *YAP‐LKD* mice in different groups were collected. HE staining and fluorescent Tunel staining was analysed microscopically (*n* = 6), Scale bar = 200 μm. The area circled by black dashed line represent injured area, Scale bar = 200 μm. (J) Suzuki score and necrosis area were examined. (K) Serum levels of ALT and AST in WT mice and *YAP‐LKD* mice were determined (*n* = 6).

To elucidate the role of LATS1 in HIRI, we employed adeno‐associated virus vectors to upregulate LATS1 expression in murine liver tissue. The findings indicated that the upregulated expression of LATS1 in liver tissue markedly diminished the protective efficacy of KYNA against HIRI in murine models (Figure 6A–D). Consistent results were obtained from cell‐based assays, where flow cytometric analysis of THLE‐2 cells revealed that LATS1 overexpression significantly elevated the apoptosis rate following H/R in hepatocytes (Figure 6E,F). Additionally, experiments conducted on primary mouse hepatocytes corroborated these observations, demonstrating that increased LATS1 expression significantly augmented the number of apoptotic hepatocytes (Figure 6G). WB analysis of primary mouse hepatocytes corroborated these findings, demonstrating a significant upregulation in the expression of pro‐apoptotic proteins BAX and cleaved caspase‐3 following the increased expression of LATS1, alongside a notable downregulation of the anti‐apoptotic protein BCL2 (Figure 6H). To further elucidate the role of YAP in mitigating HIRI, we developed a liver‐specific *YAP‐LKD* mouse model. The results indicated that YAP knockdown markedly attenuated the protective effects of KYNA against liver injury (Figure 6I–K).

### 3.7 Increased KYNA Levels in the Blood Negatively Correlate With HIRI Post‐Liver Transplantation in Patients

**FIGURE 7:**
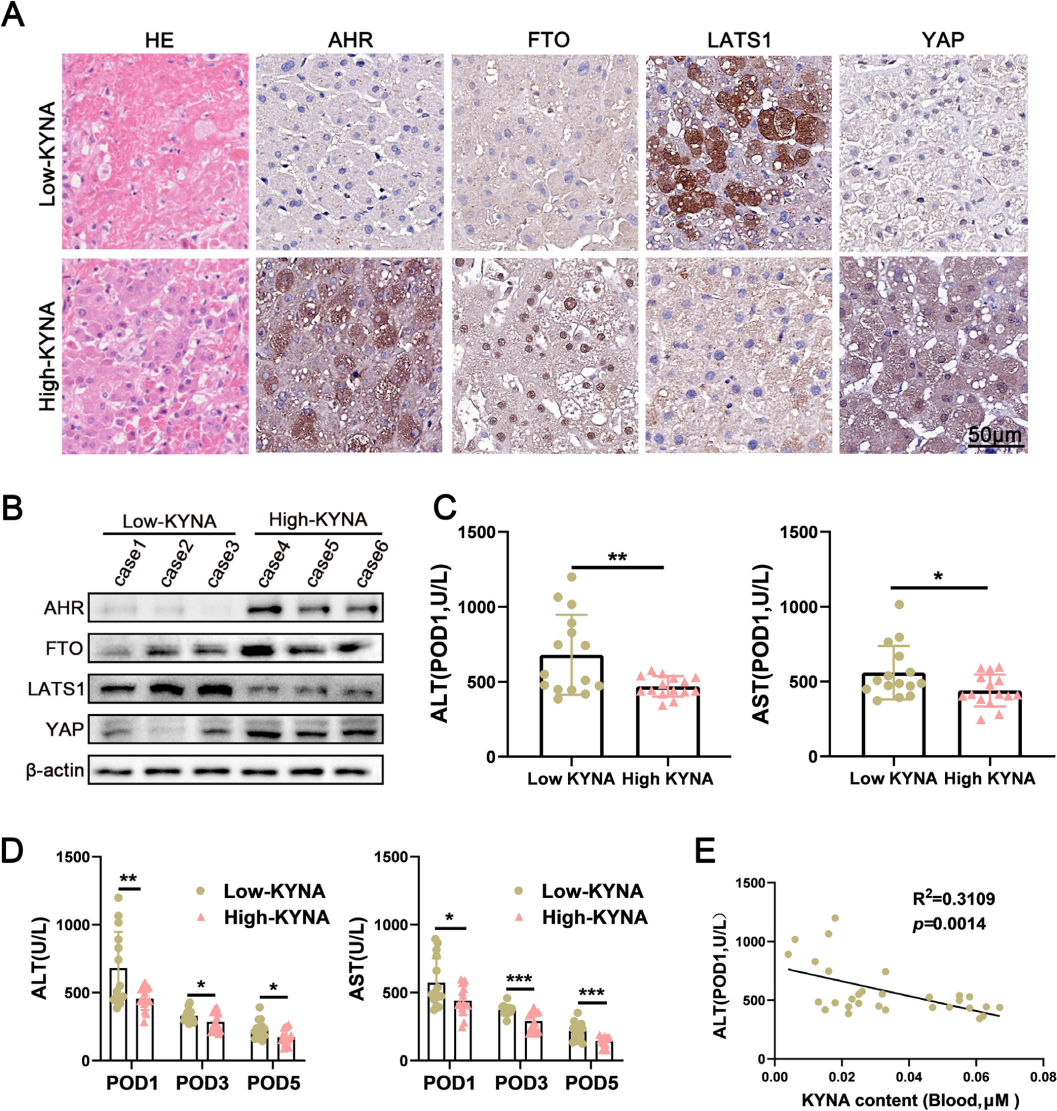
Increased KYNA levels in the blood negatively correlates with HIRI post‐liver transplantation in patients. (A) Samples of liver graft tissues (*n* = 30) from orthotopic liver transplant patients were collected. The expression of AHR, FTO, LAS1 and YAP were determined by immunohistochemical staining in Low‐KYNA group and High‐KYNA group, Scale bar = 50 μm. (B) The expression of AHR, FTO, LAS1, and YAP in liver graft tissues in Low‐KYNA group and High‐KYNA group were determined by WB analysis. (C) The correlation between ALT, AST levels at POD1 and KYNA content was examined (*n* = 15). (D) Serum ALT and AST levels between Low‐KYNA group and High‐KYNA group at each time point postoperatively were analysed (*n* = 15). (E) The correlation between ALT levels at POD1 and KYNA content was examined (*n* = 15).

Based on the aforementioned in vivo and in vitro studies, we hypothesise that KYNA is significantly associated with HIRI in humans. To evaluate this hypothesis in a clinical context, we obtained liver transplant tissue samples from 30 orthotopic liver transplant patients. These samples were subsequently categorised into high and low KYNA groups according to the patients' serum KYNA levels measured on the first postoperative day. The results of WB and Immunohistochemistry analyses indicated that the expression levels of AHR, FTO, and YAP were significantly elevated in the high KYNA group compared to the low KYNA group, whereas the expression levels of LATS1 were significantly reduced (Figure 7A,B). Furthermore, we examined the correlation between KYNA levels and the extent of liver injury in liver grafts. Liver function assessments revealed that the high KYNA group exhibited less liver function impairment and a markedly accelerated recovery rate relative to the low KYNA group (Figure 7C,D). Linear regression analysis demonstrated a negative correlation between KYNA content and postoperative liver function (Figure 7E). Consequently, KYNA content was inversely associated with liver injury following liver transplantation, suggesting its potential utility as a biomarker for IRI in this context.

## 4 Discussion

KYNA, an endogenous metabolite derived from tryptophan via the Kynurenine pathway, has been implicated in various neurophysiological and neuropathological processes. Recent advancements in scientific research have elucidated that KYNA, acting as an agonist for GPR35 and AHR, can modulate associated signalling pathways to exert anti‐inflammatory and immunomodulatory effects. In this study, we demonstrated that KYNA exerts a significant protective effect against HIRI, with this effect being partially mediated by the AHR. Mechanistically, KYNA enhances the expression of the demethylase FTO through AHR activation, thereby attenuating the release of inflammatory mediators and reducing hepatocyte apoptosis by modulating the methylation status of the Hippo signalling pathway. Furthermore, the negative correlation between serum KYNA levels and hepatic enzyme levels was substantiated.

KYNA functions as a noncompetitive antagonist of neuronal ionotropic glutamate receptors (iGLU‐R) and α7 nicotinic acetylcholine receptors (α7nAChR), exhibiting neuroprotective and other functional properties [20]. Previous research has concentrated on KYNA's role in the physiology and pathology of the central nervous system. However, recent studies have identified KYNA's significant protective effects in IRI in solid organs, mediated through GPR35 and AHR [8, 9, 21, 22]. Luc Colas et al. identified an upregulation in the expression of KYNA and tryptamine in the urine of patients exhibiting spontaneous tolerance to kidney transplantation. The authors proposed that this spontaneous tolerance may be associated with the indoleamine 2,3‐dioxygenase (IDO), GPR35, and AHR signalling pathways, as well as metabolites such as indole alkaloids [23]. Furthermore, Wyant et al. demonstrated that the activation of GPR35 is both necessary and sufficient for the ischemic protection conferred by KYNA in IRI of the rabbit heart. GPR35 interacts with KYNA to activate Gi‐ and G12/13‐coupled signalling pathways, translocates to the outer mitochondrial membrane, and associates with ATP synthase inhibitor subunit 1 (ATPIF1) to promote the formation of ATP synthase dimers, thereby mitigating ATP depletion during ischemia [24]. Despite these observations, the role of KYNA in HIRI remains inadequately investigated, and its potential implications are yet to be elucidated. The research conducted by Marciniak et al. demonstrated that KYNA exerts a protective effect against thioacetamide‐induced acute liver injury in rats, mitigates liver morphological abnormalities, and ameliorates indicators of liver function impairment, thereby advocating for the potential therapeutic application of KYNA in the treatment of acute liver failure [25]. In alignment with these findings, our study indicates that KYNA significantly attenuates HIRI by activating AHR, which in turn reduces hepatocyte apoptosis and the infiltration of inflammatory mediators.

N6‐methyladenosine methylation, the most prevalent internal mRNA modification in eukaryotic cells, plays a critical role in various physiological functions of the liver as well as in the pathogenesis of numerous liver diseases [26, 27, 28]. Recent studies have demonstrated that m6A can modulate IRI in the liver through multiple mechanisms. Specifically, the m6A methyltransferase METTL3 has been shown to inhibit hepatocyte apoptosis by upregulating the expression of heme oxygenase‐1 (HO‐1) [29]. Additionally, METTL3 may confer protection against I/R‐induced liver damage by inhibiting the phosphorylation of c‐Jun N‐terminal kinase (JNK) and extracellular signal‐regulated kinase (ERK) [30]. The m6A demethylase FTO exerts hepatoprotective effects by diminishing the methylation modification of Dynamin‐related protein 1 (Drp1) mRNA, thereby inhibiting Drp1‐mediated mitochondrial fragmentation [16]. Additionally, FTO decreases the stability of Acyl‐CoA synthetase long‐chain family member 4 (Acsl4) and transferrin receptor 1 (Tfrc) mRNA in an m6A‐dependent manner, mitigating IRI in the aging liver by reducing ferroptosis [31]. Despite these findings, the precise mechanisms through which m6A methylation regulates HIRI remain unclear. In this study, we discovered that KYNA upregulates the demethylase FTO via AHR mediation, ultimately leading to a reduction in m6A methylation levels. Therefore, the question arises as to whether KYNA can mitigate HIRI by upregulating the expression of FTO. A series of functional gain and loss experiments have demonstrated that KYNA can effectively alleviate IRI in the liver through the upregulation of FTO expression.

As the first identified demethylase, FTO plays a crucial role in the regulation of m6A methylation. To the best of our knowledge, the precise biological function of FTO in the context of organ IRI remains unknown. Previous research has demonstrated that the overexpression of FTO significantly diminishes m6A methylation levels post‐stroke, mitigates damage to both grey and white matter, and enhances the recovery of motor function, cognitive abilities, and depression‐like behaviors [32]. Furthermore, FTO has been shown to alleviate myocardial IRI by inhibiting Cbl proto‐oncogene, E3 ubiquitin protein ligase (Cbl)‐induced β‐catenin ubiquitination and degradation, and suppressing NLR family pyrin domain containing 3 (NLRP3)‐mediated pyroptosiss [33]. In this study, we observed that the upregulation of FTO led to a reduction in the methylation modification level of LATS1 mRNA, resulting in decreased stability of both the mRNA and its corresponding protein expression. This, in turn, facilitated the nuclear translocation of YAP, thereby mitigating hepatocyte apoptosis and HIRI. LATS1, a central kinase in the Hippo signalling pathway, plays a critical role in regulating cell proliferation, apoptosis, and stem cell self‐renewal through its modulation of YAP phosphorylation and nuclear translocation [34, 35, 36]. To date, the regulatory mechanisms governing LATS1 in disease contexts remain incompletely understood, underscoring the necessity of investigating targeted therapeutic strategies. LATS1, a pivotal kinase within the Hippo signalling pathway, has been demonstrated to significantly influence the regulation of various tumour growths [37, 38, 39]. Notably, circular RNA XRN2 (circXRN2) has been shown to inhibit histone H3 lysine 18 acetylation (H3K18ac)‐driven tumour progression in human bladder cancer by activating the Hippo signalling pathway through the prevention of LATS1 degradation [40]. The suppression of METTL3 or YTHDF2 protein expression has been shown to elevate LATS1 mRNA levels and inhibit breast cancer progression through the activation of YAP/TAZ within the Hippo pathway [41]. Speckle‐type POZ protein (SPOP) has been identified to specifically interact with LATS1, facilitating its polyubiquitination and subsequent degradation, thereby enhancing the invasiveness of kidney cancer cells [42]. The majority of these investigations have focused on elucidating the mechanistic role of LATS1 in tumourigenesis. Additionally, numerous studies have demonstrated a significant association between the Hippo signalling pathway and liver regeneration following injury. As a principal effector within the Hippo signalling pathway, YAP is integral to the regulation of organ size and volume, tissue regeneration, and the protection against organ damage [43, 44]. Our previous research has elucidated that the protective effects of YAP on HIRI are significantly contingent upon the activation of autophagy. This process mitigates apoptosis by diminishing mitochondrial reactive oxygen species (ROS) production and preserving mitochondrial membrane integrity. Furthermore, the JNK signalling pathway serves as a critical mediator of YAP‐induced autophagy and is modulated through YAP‐TEAD complex formation [45]. In the present study, we observed that KYNA markedly influences the expression and functionality of LATS1 and YAP by upregulating the expression of FTO, thereby exerting a protective effect against HIRI. Notably, the protective efficacy of these interventions was significantly diminished following LATS1 overexpression, indicating that LATS1 is pivotal in KYNA‐mediated hepatoprotection.

This study has certain limitations. KYNA enhances the expression of AHR by activating it, leading to its translocation from the cytoplasm to the nucleus. Within the nucleus, AHR influences the methylation modification of the Hippo signalling pathway by promoting the expression of FTO, thereby exerting a protective effect in HIRI. However, further research is required to elucidate the specific mechanisms through which KYNA regulates FTO expression via AHR activation in the context of HIRI. Furthermore, clinical studies have demonstrated an inverse correlation between KYNA levels and the severity of liver injury post‐transplantation. However, the precise role of KYNA remains ambiguous, necessitating a comprehensive clinical trial to elucidate its protective effects on liver function following transplantation. Our findings provide substantial evidence that KYNA enhances demethylase FTO activity via AHR mediation, thereby mitigating HIRI by downregulating LATS1 expression and facilitating YAP nuclear translocation. Our study establishes a foundational basis for the further investigation of KYNA's role in mitigating organ IRI and proposes a strategic framework for the identification of pharmacological and molecular targets aimed at the prevention and treatment of HIRI.

## Author Contributions

Contributions: (I) Conception and design: WJ Zheng, WX Wang, HQ Chen, SG Zhu, H Li; (II) Administrative support: P Zhang, WF Zhu, SG Zhu, H Li; (III) Provision of study materials or patients: WX Wang, WJ Zheng, HQ Chen, Y Zhang; (IV) Collection and assembly of data: XJ Yan, Y Yang; (V) Data analysis and interpretation: WX Wang, WJ Zheng, HQ Chen, KM He; (VI) Manuscript writing: All authors; (VII) Final approval of manuscript: All authors.

## Ethics Statement

Ethical approval for the animal experiments was granted by the laboratory animal care and ethics committee of Guangdong Laidi Biomedical Research Institute Co., LTD (approval number: 2024036–1). The clinical study was approved by the Ethics Committee of the Third Affiliated Hospital of Sun Yat‐sen University (approval number: 2024–267‐01, dated September 26, 2024) and conducted in accordance with the principles of the Declaration of Helsinki.

## Consent

The authors have nothing to report.

## Conflicts of Interest

The authors declare no conflicts of interest.

## Supporting information


**Figure S1.** (A) Mice were administered KYNA at concentrations of 0 mg/kg, 10 mg/kg, 20 mg/kg, and 40 mg/kg via abdominal cavity injection for a duration of 2 weeks. Liver samples were collected from different treated mice. HE staining and fluorescent TUNEL staining was analysed microscopically (*n* = 6). The area circled by black dashed line represent injured area. Scale bar = 200 μm. (B) Suzuki score and necrosis area were examined. (C) The expression of AHR and GPR35 with different treatment mice were determined by WB analysis. (D) RNA level of AHR and GPR35 with different treatment mice was examined by RT‐qPCR (*n* = 3). (E) m6A dot blot assessed m6A mRNA methylation of mice and THLE‐2 cells with different treatment. (F) The representative TUNEL staining was microscopically analysed in primary mouse hepatocytes isolated from mice with different treatments. (G) The expression of BCL2, cleaved caspase‐3 and BAX in primary mouse hepatocytes isolated from mice in different groups were determined by WB analysis. (H) m6A dot blot assessed m6A mRNA methylation of mice with different treatment. (I) PCA analysis of the differential expression profiles between the H/*R* + DMSO group and the H/*R* + KYNA group. (J) GO enrichment analysis correlated with different m6A peak genes.

**Figure S2.** (A) RNA level of LATS1 in H/*R* + DMSO group and the H/*R* + KYNA group was examined by RT‐qPCR (*n* = 3). (B) Protein level of LATS1, p‐LATS1 in H/*R* + DMSO group and the H/*R* + KYNA group was examined by WB. (C) The expression YAP and p‐YAP of cytosolic and nuclear‐enriched fractions in THLE‐2 cells in H/*R* + DMSO group and the H/*R* + KYNA group was examined by WB analysis. β‐actin and Histone 3 were used as loading controls of cytosolic and nuclear fractions respectively. (D) Immunofluorescence staining of LATS1 and YAP in THLE‐2 cells. Nuclei were counterstained with DAPI, Scale bar = 50 μm. (E) Relative quantification of IF staining of LATS1 and YAP in THLE‐2 cells. (F) Potential modification sites in LATS1 mRNA region. (G, H) Effects of YTHDF2 on the expression of LATS1 in THLE‐2 cells were determined by WB analysis.

**Data S1.** Supporting Information.

**Table S1.** Primer sequences used for RT‐qPCR analysis.

**Table S2.** Effects of KYNA on physiological functions and surgical stress tolerance in mice.

## Data Availability

The datasets used and/or analysed during the current study are available from the corresponding author upon reasonable request.
