# Supplementary figures and images for: KYNA Ameliorates Hepatic Ischemia–Reperfusion Injury by Activating the Hippo Signalling Pathway via FTO‐Dependent m6A Demethylation of LATS1
Source: Cell Prolif. 2025 Apr 25;58(10):e70048. doi: 10.1111/cpr.70048 (PMC12508694; doi:10.1111/cpr.70048)

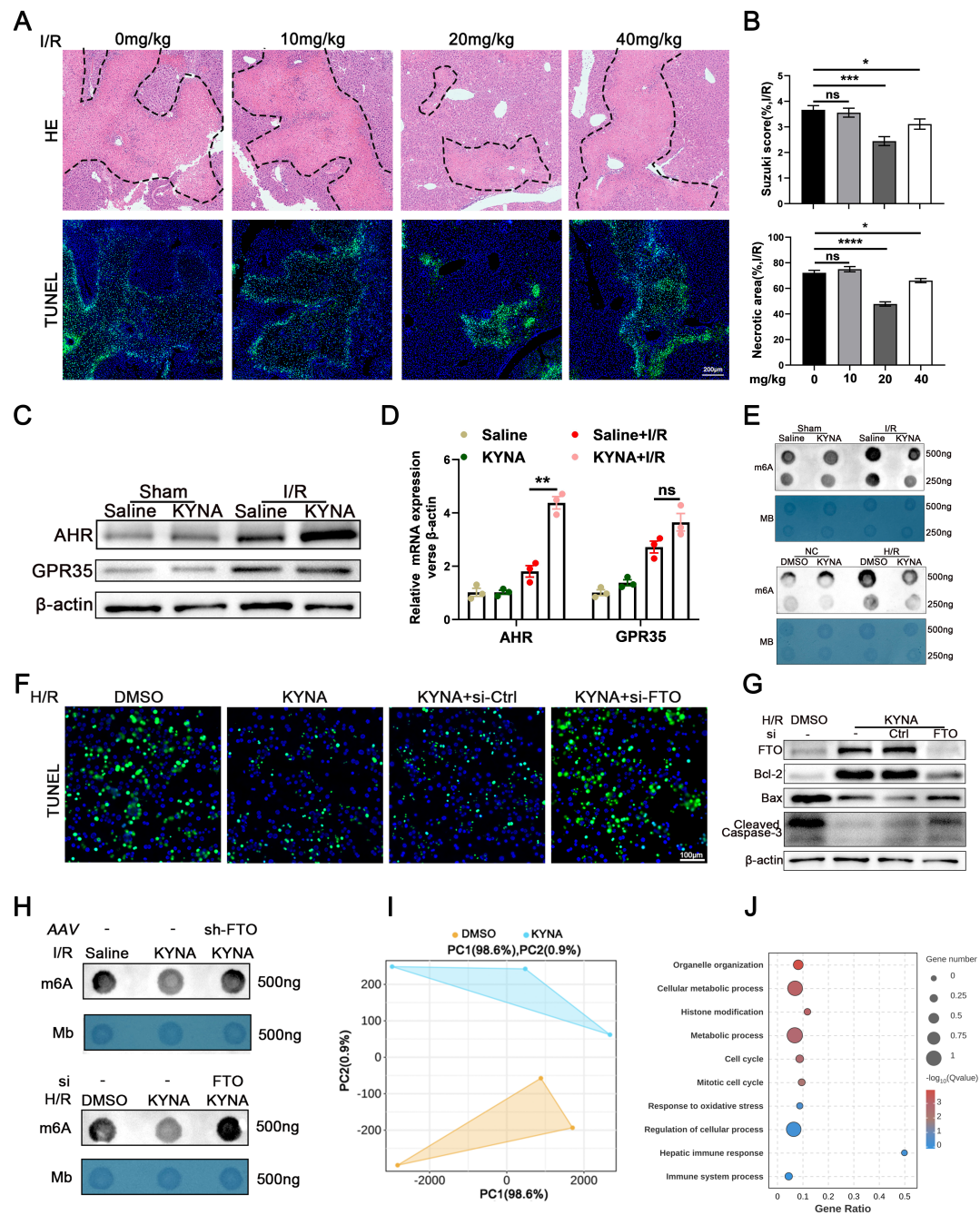

Supplement: Supplementary file 1 — Figure S1. (A) Mice were administered KYNA at concentrations of 0 mg/kg, 10 mg/kg, 20 mg/kg, and 40 mg/kg via abdominal cavity injection for a duration of 2 weeks. Liver samples were collected from different treated mice. HE staining and fluorescent TUNEL staining was analysed microscopically (n = 6). The area circled by black dashed line represent injured area. Scale bar = 200 μm. (B) Suzuki score and necrosis area were examined. (C) The expression of AHR and GPR35 with different treatment mice were determined by WB analysis. (D) RNA level of AHR and GPR35 with different treatment mice was examined by RT‐qPCR (n = 3). (E) m6A dot blot assessed m6A mRNA methylation of mice and THLE‐2 cells with different treatment. (F) The representative TUNEL staining was microscopically analysed in primary mouse hepatocytes isolated from mice with different treatments. (G) The expression of BCL2, cleaved caspase‐3 and BAX in primary mouse hepatocytes isolated from mice in different groups were determined by WB analysis. (H) m6A dot blot assessed m6A mRNA methylation of mice with different treatment. (I) PCA analysis of the differential expression profiles between the H/R + DMSO group and the H/R + KYNA group. (J) GO enrichment analysis correlated with different m6A peak genes. [file CPR-58-e70048-s002.pdf]

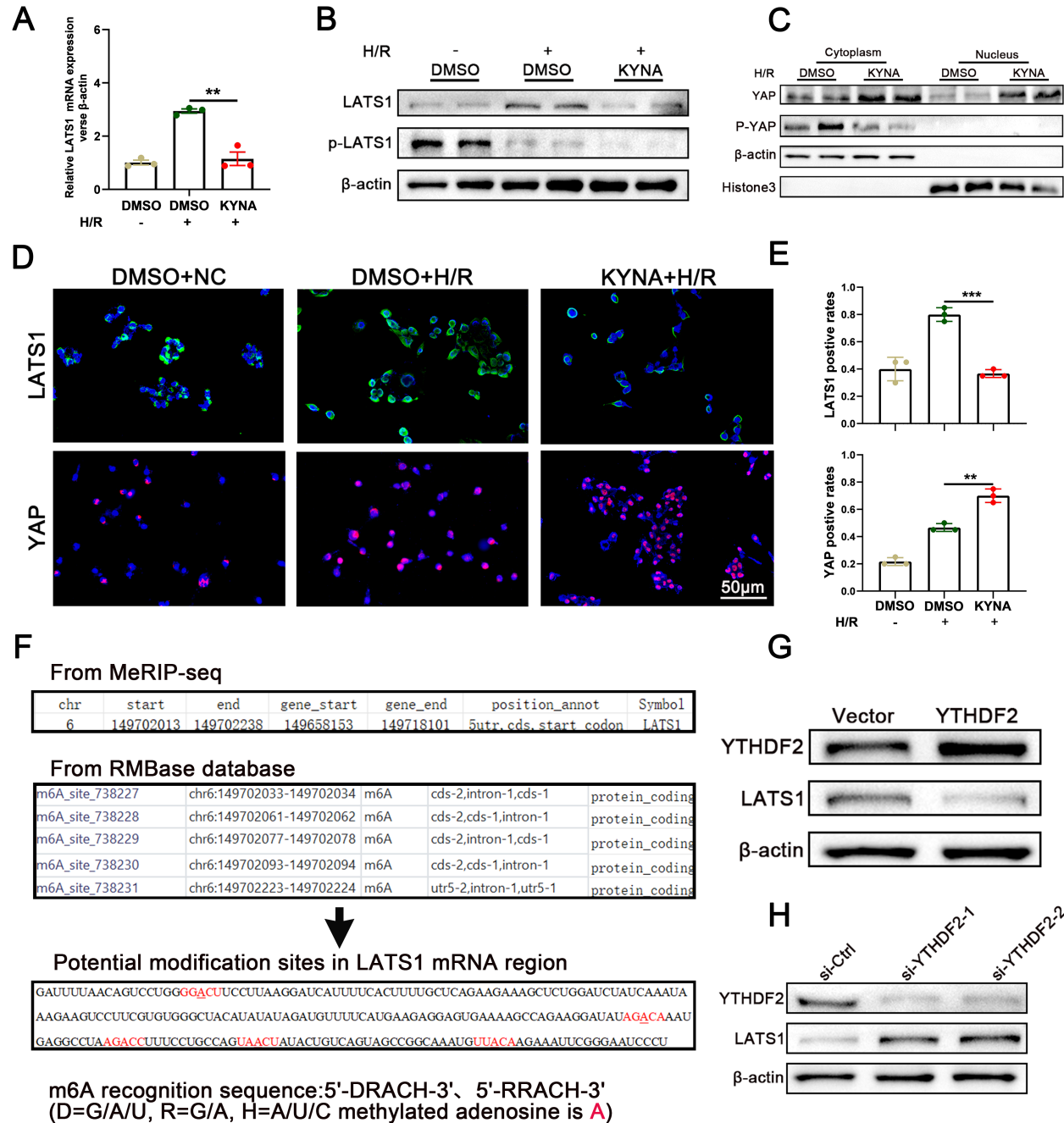

Supplement: Supplementary file 2 — Figure S2. (A) RNA level of LATS1 in H/R + DMSO group and the H/R + KYNA group was examined by RT‐qPCR (n = 3). (B) Protein level of LATS1, p‐LATS1 in H/R + DMSO group and the H/R + KYNA group was examined by WB. (C) The expression YAP and p‐YAP of cytosolic and nuclear‐enriched fractions in THLE‐2 cells in H/R + DMSO group and the H/R + KYNA group was examined by WB analysis. β‐actin and Histone 3 were used as loading controls of cytosolic and nuclear fractions respectively. (D) Immunofluorescence staining of LATS1 and YAP in THLE‐2 cells. Nuclei were counterstained with DAPI, Scale bar = 50 μm. (E) Relative quantification of IF staining of LATS1 and YAP in THLE‐2 cells. (F) Potential modification sites in LATS1 mRNA region. (G, H) Effects of YTHDF2 on the expression of LATS1 in THLE‐2 cells were determined by WB analysis. [file CPR-58-e70048-s005.pdf]
