# Supplementary material for: KYNA Ameliorates Hepatic Ischemia–Reperfusion Injury by Activating the Hippo Signalling Pathway via FTO‐Dependent m6A Demethylation of LATS1
Source: Cell Prolif. 2025 Apr 25;58(10):e70048. doi: 10.1111/cpr.70048 (PMC12508694; doi:10.1111/cpr.70048)
Supplement: Supplementary file 3 — Data S1. Supporting Information. [file CPR-58-e70048-s004.docx]

**Establishment of the mouse HIRI model**

A murine model of 70% hepatic ischemia-reperfusion (I/R) injury was established using male C57BL/6J mice, aged six weeks and weighing between 20 and 25 grams. In this model, partial ischemia was induced by occluding 70% of the portal triad, which includes the portal vein, hepatic artery, and bile duct, for 90 minutes. Following a reperfusion period of 12 hours, the mice were euthanized, and liver tissues along with blood samples were collected for subsequent analysis. A sham control group underwent the same surgical procedure without the occlusion of blood vessels.

**Animal adeno-associated virus-8 (AAV8) injection and processing**

An adeno-associated virus serotype 8 (AAV8) vector was employed to construct gene knockdown and overexpression vectors targeting AHR, FTO, and LATS1 in the liver, provided by GeneChem (Shanghai, China). The recombinant virus, at a concentration of 1.5×10^11 viral genomes, was diluted in 0.2 mL of normal saline solution and administered via tail vein injection in mice. After 3 weeks, WB was employed to assess the efficiency of knockdown or overexpression. Subsequently, the HIRI model was established.

**Histology, immunohistochemistry and immunofluorescence staining**

Liver tissues were fixed in 4% paraformaldehyde solution, embedded in paraffin, sections were stained for HE, and light microscopy was used for histology. The degree of liver injury was assessed using Suzuki’s score and necrotic area assessment. Immunohistochemistry and immunofluorescence staining were performed according to the manufacturer's instructions. The Primary antibodies used are AHR, LATS1, and FTO (Proteintech, Rosemont, IL, USA) and YAP (Cell Signaling Technology, Danvers, MA, USA).

**Liver function and inflammatory cytokine assays**

Levels of alanine aminotransferase (ALT) and aspartate aminotransferase (AST) were measured using an automated analyzer (Hitachi, Tokyo, Japan) according to the manufacturer's instructions. Serum levels of tumor necrosis factor-α (TNF-α) and interleukin-6 (IL-6) were determined using a mouse enzyme-linked immunosorbent assay (ELISA) kit (Shenzhen New Biotechnology Co., Ltd., Shenzhen, Guangdong, China).

**Isolation and identification of primary mouse hepatocytes**

Primary mouse hepatocytes were isolated from C57BL/6 mice by portal vein perfusion. The mice were anesthetized and sterilized, and the abdominal cavity was opened to fully expose the portal vein and inferior vena cava. After portal catheterization, calcium- and magnesium-deficient Hank Equilibrium salt solution (HBSS) was infused. Subsequently, the infusion solution was switched to a solution containing calcium and magnesium, and the liver was digested with 1 mg/mL H-type collagenase at a constant temperature of 37°C and an infusion rate of 8 mL/min. When a loose mesh appears, the liver is quickly removed, shredded in a pre-cooled complete medium, filtered through a 70µm cell filter and centrifuged to obtain cell precipitates. primary mouse hepatocytes were purified by gradient centrifugation using 90% Percoll solution. The isolated primary hepatocytes were cultured in complete medium with 1% penicillin streptomycin and 100 nM dexamethasone added to DMEM/Ham’s F-12 .

**Flow cytometry analysis**

For flow cytometry analysis, THLE2 cells were stained using the Beyotime Annexin V-FITC Apoptosis Detection Kit, and flow cytometry was conducted with CytoFLEX SRT cytometry, with subsequent analysis performed using CytoFLEX software.

**Western blot**

Freshly isolated human mouse liver tissue, PMHs and THLE2 cells were lysed with protease inhibitor RIPA buffer. After centrifugation, the protein concentration in the supernatant was determined with the BCA Protein Detection Kit. Proteins (50 μg) were separated with SDS-PAGE on a 10% or 12% gel and then transferred to a PVDF membrane. Membranes were closed with 5% skim milk and treated with anti-BCL2, anti-BAX, anti-cleaved caspase-3, anti-β-actin, anti-Histon-3, anti-AHR, anti-p-YAP, anti-YAP, anti-FTO, anti-p-LATS1, anti-LATS1, anti-YTHDF1, anti-YTHDF2, (Cell Signaling Technology, Danvers, MA, USA). After washing, the membrane was incubated with secondary antibody for 1 h at room temperature. Finally, after using an enhanced chemiluminescent (ECL) substrate, the blot was investigated with a FluorChem Systems imager (ProteinSimple, CA, USA) and analyzed by Image J. For AHR/FTO co-immunoprecipitation, lysed cells are pre-cleared with 30μL protein G agarose beads (Santa Cruz Biotech, USA) for 1 hour, overnight with AHR antibodies or FTO antibodies at 4°C, then protein G agarose beads are added and incubated at 4°C for 3 hours. The immune complex was washed 5 times, resuspended in a 3x SDS-PAGE loading buffer, boiled for 10 min and then immunoblotted with AHR and FTO.

**TUNEL staining**

Apoptotic cells in liver tissue and PMHs were detected using the TUNEL assay kit (Beyotime, Haimen, Jiangsu, China) according to the manufacturer's instructions. After dewaxing, protease K without DNA enzyme (20 μg/mL; Beyotime, Haimen, Jiangsu, China) was added to paraffin sections and incubated for 30 min at room temperature. After washing with PBS solution, TUNEL working solution was added and incubated for 1 h at 37 °C in the dark. Finally, after counterstaining with DAPI (1:500 dilution; Vector Laboratories, Burlingame, CA, USA), the samples were observed under a fluorescence microscope, and TUNEL-positive cells were detected.

**Dot blot**

After isolating total RNA from in vitro and in vivo samples with TRIzol, RNA samples were diluted to 250 ng with nuclease-free water and then denatured at 95°C for 5 minutes. The samples were then transferred onto Amersham Hybond-N+ membranes (GE Healthcare, USA). After two rounds of 2400 UV crosslinking (5 min each), the membrane was washed with 0.1% TBST (DEPC water complex), blocked with 0.1% TBST in 5% skim milk, and mixed with anti-m6A antibody (1:1000; Cell Signaling Technology, CA, USA) and incubated overnight at 4°C. After washing with 0.1% TBST, the membranes were incubated for 1 h at room temperature. Finally, they were observed with the ECL Western Blotting Detection Kit (201005-79, Advansta, CA, USA). To determine the total amount of input RNA, membranes were stained with 0.02% methylene blue (M4159, Sigma-Aldrich, USA) in 0.3 M sodium acetate (pH 5.2) prior to blocking. Density analysis was performed with MB normalized m6A (ImageJ Software, United States).

**Dual-luciferase reporter assay**

THLE2 cells were seeded in a 6-well plate and cultured overnight. Subsequently, plasmids containing either the wild-type LATS1 or a mutant variant, in which the five putative m6A motifs were simultaneously mutated at the m6A locus, were co-transfected into THLE2 cell lines. This was performed in conjunction with siFTO or siCtrl using Lipofectamine 3000 reagent. Following a 48-hour incubation period post-transfection, cells were harvested for a luciferase assay. Firefly luciferase activity was measured and normalized to Renilla luciferase activity utilizing a dual-luciferase reporter system.

**Methylated RNA immunoprecipitation (MeRIP)**

The MeRIP assay was performed using the riboMeRIP m6A Transcriptome Analysis Kit (C11051-1, RiboBio, Guangzhou, China) to assess the m6A modification of LATS1 mRNA. After treating THLE2 cells with DMSO or KYNA, at least 100 μg of total RNA was extracted with TRIzol Reagent. After RNA quantification, 100 μg of total RNA was randomly split into fragments of 200 or fewer nucleotides at 70 °C, and the fragment RNA was precipitated overnight. One-tenth of the RNA was saved as an input control. The beads were incubated with anti-m6A antibody or IgG antibody for 4 h at 4 °C with rotation. After three washes, the beads, RIP immunoprecipitation buffer, and fragment RNA were mixed and incubated overnight at 4 °C with rotation to finally purify the input and RIP samples. The enrichment of m6A was further analyzed by RT-qPCR.

**Methylated RIP-sequencing (MeRIP-seq) and RNA-sequencing (RNA-seq)**

MeRIP-seq and RNA-seq were completed by Gene Denovo Biotechnology Co., Ltd (Guangzhou, China). Briefly, THLE2 cells were treated with DMSO and KYNA, followed by hypoxic reoxygenation. Total RNA was extracted from cells for mRNA sequencing and m6A sequencing. For m6A sequencing, approximately 30 μg of total RNA was chemically fragmented into 100-nt-long fragments. Approximately 1/10 of the RNA fragments were isolated as input controls for further RNA sequencing. The remaining fragment RNA was premixed with anti-m6A antibody in IP buffer [50 mM Tris-HCl, 750 mM NaCl, and 0.5% Igepal CA-630] overnight at 4 °C. The eluted RNA was precipitated with 75% ethanol. The unprocessed input control fragment (IP) was converted into the final cDNA library. The average insertion size of the peer library was ~150 bp. 2×150 bp paired-end sequencing was performed on the Illumina NovaSeq 6000 platform. For RNA-Seq, eukaryotic mRNA with polyA tails was enriched by magnetic beads with Oligo(dT), and the mRNA was interrupted with buffer. The first strand of cDNA was synthesized in the M-MuLV reverse transcriptase system using fragmented mRNA as a template and random oligonucleotides as primers, followed by RNase H degradation of the RNA strand, and the second strand of cDNA was synthesized with dNTPs as raw material under the DNA polymerase I system. After purifying the double-stranded cDNA, the cDNA was repaired at the end, A-tailed, and ligated with sequencing adapters. About 200 bp of cDNA was screened with AMPure XP beads, PCR amplification was performed, and the PCR product was purified again with AMPure XP beads to obtain the library. Sequencing was then performed to generate a 150 bp paired-end sequence. The reads of each sample were aligned with the reference genome, and the number of reads for each gene was calculated. Differentially expressed genes were analyzed by DESeq2 software using a false discovery rate (FDR) of < 0.05. Gene ontology (GO) enrichment analysis of differentially expressed genes was performed using the GOseq R software package.
